# 4-[(2-Hydr­oxy-1-naphth­yl)(piperidin-1-yl)meth­yl]benzonitrile

**DOI:** 10.1107/S1600536809033728

**Published:** 2009-08-29

**Authors:** Yuan Zhang, Yong Hua Li

**Affiliations:** a Ordered Matter Science Research Center, College of Chemistry and Chemical, Engineering, Southeast UniVersity, Nanjing 211189, People’s Republic of China

## Abstract

In the title compound, C_23_H_22_N_2_O, obtained from the condensation reaction of 4-formyl­benzonitrile, 2-naphthol and piperidine, the dihedral angle between the naphthalene ring system and the benzene ring is 75.31 (4)°. The piperidine ring adopts a chair conformation. The crystal structure is stabilized by inter­molecular C—H⋯N hydrogen bonds, which link the mol­ecules into centrosymmetric dimers. An intra­molecular O—H⋯N hydrogen bond is also present.

## Related literature

For applications of Betti-type reactions, see: Zhao & Li *et al.* (2004 ▶); Lu *et al.* (2002 ▶); Xu *et al.* (2004 ▶); Wang *et al.* (2005 ▶)
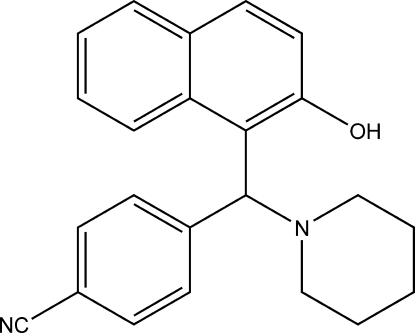


## Experimental

### 

#### Crystal data

C_23_H_22_N_2_O
*M* *_r_* = 342.43
Monoclinic, 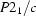

*a* = 6.9989 (6) Å
*b* = 15.588 (1) Å
*c* = 17.211 (1) Å
β = 101.207 (2)°
*V* = 1841.9 (3) Å^3^
*Z* = 4
Mo *K*α radiation
μ = 0.08 mm^−1^
*T* = 296 K
0.2 × 0.1 × 0.1 mm

#### Data collection

Rigaku SCXmini diffractometer
Absorption correction: multi-scan (*CrystalClear*, Rigaku, 2005 ▶) *T*_min_ = 0.98, *T*_max_ = 0.98
10945 measured reflections
3245 independent reflections
2661 reflections with *I* > 2σ(*I*)
*R*_int_ = 0.023

#### Refinement

*R*[*F* ^2^ > 2σ(*F* ^2^)] = 0.040
*wR*(*F* ^2^) = 0.108
*S* = 1.05
3245 reflections
227 parameters
H-atom parameters constrained
Δρ_max_ = 0.15 e Å^−3^
Δρ_min_ = −0.13 e Å^−3^

### 

Data collection: *CrystalClear* (Rigaku, 2005 ▶); cell refinement: *CrystalClear*; data reduction: *CrystalClear*; program(s) used to solve structure: *SHELXS97* (Sheldrick, 2008 ▶); program(s) used to refine structure: *SHELXL97* (Sheldrick, 2008 ▶); molecular graphics: *SHELXTL/PC* (Sheldrick, 2008 ▶) and *DIAMOND* (Brandenburg, 1998 ▶); software used to prepare material for publication: *SHELXTL/PC*.

## Supplementary Material

Crystal structure: contains datablocks I, global. DOI: 10.1107/S1600536809033728/lx2110sup1.cif

Structure factors: contains datablocks I. DOI: 10.1107/S1600536809033728/lx2110Isup2.hkl

Additional supplementary materials:  crystallographic information; 3D view; checkCIF report

## Figures and Tables

**Table 1:** Hydrogen-bond geometry (Å, °)

*D*—H⋯*A*	*D*—H	H⋯*A*	*D*⋯*A*	*D*—H⋯*A*
O1—H1⋯N2	0.99	1.70	2.614	151
C14—H14⋯N1^i^	0.93	2.55	3.395 (2)	151

Symmetry code: (i) 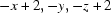.

**Fig. 1.**
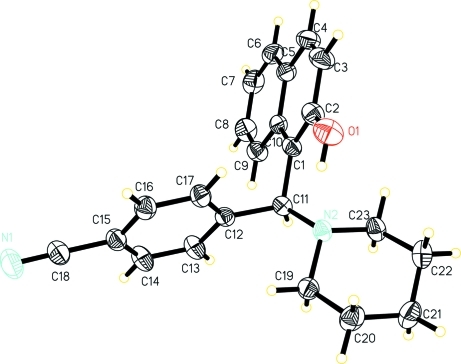
Perspective structure of the title compound, showing the atom-numbering scheme. Displacement ellipsoids are drawn at the 30% probability level.

**Fig. 2.**
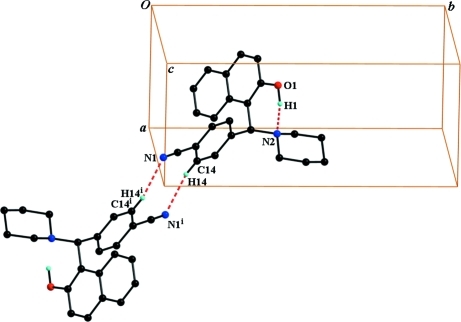
C–H···N and O–H···N hydrogen bonds (dotted lines) in the crystal structure of the title compound. [Symmetry code : (i) - x + 2, - y, - z + 2.]

## supplementary crystallographic information

### Comment

Over one hundred years ago, Betti developed a straightforward synthesis involving the condensation of 2-naphthol, ammonia and equivalents of benzaldehyde, followed by the addition of HCl and KOH to yield 1-(a-aminobenzyl)-2-naphthol. This product which possesses an asymmetric carbon center is known as a Betti base (Zhao & Li *et al.* 2004). Betti-type reaction is an important method to synthesize chiral ligands and by this method many unnatural homochiral amino-phenol compounds have been obtained (Lu *et al.* 2002; Xu *et al.* 2004; Wang *et al.* 2005). Here we report the synthesis and crystal structure of the title compound, 4-[(2-hydroxy-1-naphthyl)(1-piperidinyl)methyl]benzonitrile (Fig. 1).

The naphthalene (A; C1-C10), benzene (B; C12-C17) and piperidine (C; N2/C19-C23) rings are planar and the dihedral angles between A/B, A/C, and B/C are 75.31 (4)°, 67.24 (5)°, and 88.80 (5)°, respectively. The crystal structure (Fig. 2) is stabilized by intermolecular C–H···N hydrogen bonds between an H atom of benzene ring and the N atom of the nitrile group, with a C14–H14···N1i (Table 1 and Fig. 2), which link the molecules into centrosymmetric dimers. In addition, the crystal structure exhibits an intramolecular O–H···N hydrogen bond, with a O1–H1···N2 (Table 1 and Fig. 2).

### Experimental

4-Formylbenzonitrile  (1.97 g, 0.015 mol) and piperidine (1.275 g, 0.015 mol) was added to 2-naphthol (2.16 g, 0.015 mol) without solvent under nitrogen. The temperature was raised to 120°C in one hour gradually and the mixture was stirred at this temperature for 10 h. The system was treated with 20 ml of ethanol 95% and cooled. The precipitate was filtered and washed with a small amount of ethanol 95%. The title compound was isolated using column chromatography (Petroleum ether: ethyl acetate-4:1). Single crystals suitable for X-ray diffraction analysis were obtained from slow evaporation of a solution of the title compound in ethyl acetate at room temperature.

### Refinement

H atoms bonded to O atoms were located in a difference map and refined freely. Other H atoms were positioned geometrically and refined using a riding model, with C–H = 0.93-0.97 Å and *U*_iso_(H) = 1.3-1.6*U*_eq_(C).

### Crystal data

**Table:**

C_23_H_22_N_2_O	*F*(000) = 728
*M**_r_* = 342.43	*D*_x_ = 1.235 Mg m^−^^3^
Monoclinic, *P*2_1_/*c*	Mo *K*α radiation, λ = 0.71073 Å
Hall symbol: -P 2ybc	Cell parameters from 3760 reflections
*a* = 6.9989 (6) Å	θ = 2.1–26.0°
*b* = 15.588 (1) Å	µ = 0.08 mm^−^^1^
*c* = 17.211 (1) Å	*T* = 296 K
β = 101.207 (2)°	Prism, colorless
*V* = 1841.9 (3) Å^3^	0.2 × 0.1 × 0.1 mm
*Z* = 4	

### Data collection

**Table:**

Rigaku SCXmini diffractometer	3245 independent reflections
Radiation source: fine-focus sealed tube	2661 reflections with *I* > 2σ(*I*)
graphite	*R*_int_ = 0.023
CCD_Profile_fitting scans	θ_max_ = 26.0°, θ_min_ = 2.4°
Absorption correction: multi-scan (*CrystalClear*, Rigaku, 2005)	*h* = −6→8
*T*_min_ = 0.98, *T*_max_ = 0.98	*k* = −19→18
10945 measured reflections	*l* = −21→21

### Refinement

**Table:**

Refinement on *F*^2^	Primary atom site location: structure-invariant direct methods
Least-squares matrix: full	Secondary atom site location: difference Fourier map
*R*[*F*^2^ > 2σ(*F*^2^)] = 0.040	Hydrogen site location: difference Fourier map
*wR*(*F*^2^) = 0.108	H-atom parameters constrained
*S* = 1.05	*w* = 1/[σ^2^(*F*_o_^2^) + (0.0474*P*)^2^ + 0.2574*P*] where *P* = (*F*_o_^2^ + 2*F*_c_^2^)/3
3245 reflections	(Δ/σ)_max_ < 0.001
227 parameters	Δρ_max_ = 0.14 e Å^−^^3^
0 restraints	Δρ_min_ = −0.13 e Å^−^^3^

### Special details

**Table:**

Geometry. All esds (except the esd in the dihedral angle between two l.s. planes) are estimated using the full covariance matrix. The cell esds are taken into account individually in the estimation of esds in distances, angles and torsion angles; correlations between esds in cell parameters are only used when they are defined by crystal symmetry. An approximate (isotropic) treatment of cell esds is used for estimating esds involving l.s. planes.
Refinement. Refinement of F^2^ against ALL reflections. The weighted R-factor wR and goodness of fit S are based on F^2^, conventional R-factors R are based on F, with F set to zero for negative F^2^. The threshold expression of F^2^ > 2sigma(F^2^) is used only for calculating R-factors(gt) etc. and is not relevant to the choice of reflections for refinement. R-factors based on F^2^ are statistically about twice as large as those based on F, and R- factors based on ALL data will be even larger.

### Fractional atomic coordinates and isotropic or equivalent isotropic displacement parameters (Å^2^)

**Table:**

	*x*	*y*	*z*	*U*_iso_*/*U*_eq_	
C1	0.4754 (2)	0.29551 (10)	0.68027 (8)	0.0440 (4)	
C2	0.3079 (2)	0.33944 (11)	0.68632 (9)	0.0530 (4)	
C3	0.1365 (2)	0.32981 (13)	0.62827 (11)	0.0654 (5)	
H3	0.0261	0.3612	0.6326	0.078*	
C4	0.1307 (2)	0.27582 (13)	0.56662 (10)	0.0646 (5)	
H4	0.0163	0.2705	0.5290	0.078*	
C5	0.2951 (2)	0.22744 (11)	0.55839 (9)	0.0525 (4)	
C6	0.2914 (3)	0.16868 (12)	0.49529 (10)	0.0660 (5)	
H6	0.1773	0.1627	0.4577	0.079*	
C7	0.4480 (3)	0.12125 (12)	0.48807 (10)	0.0699 (5)	
H7	0.4413	0.0827	0.4464	0.084*	
C8	0.6191 (3)	0.13057 (12)	0.54346 (10)	0.0653 (5)	
H8	0.7275	0.0980	0.5386	0.078*	
C9	0.6308 (2)	0.18670 (10)	0.60497 (9)	0.0539 (4)	
H9	0.7479	0.1918	0.6410	0.065*	
C10	0.4703 (2)	0.23747 (10)	0.61557 (8)	0.0444 (4)	
C11	0.6626 (2)	0.30548 (9)	0.74220 (8)	0.0413 (3)	
H11	0.7723	0.2984	0.7150	0.050*	
C12	0.6784 (2)	0.23582 (9)	0.80461 (8)	0.0420 (3)	
C13	0.8360 (2)	0.18047 (11)	0.81721 (9)	0.0552 (4)	
H13	0.9315	0.1863	0.7868	0.066*	
C14	0.8543 (3)	0.11701 (11)	0.87378 (10)	0.0616 (5)	
H14	0.9603	0.0799	0.8809	0.074*	
C15	0.7144 (2)	0.10873 (10)	0.92003 (9)	0.0508 (4)	
C16	0.5568 (2)	0.16398 (10)	0.90883 (9)	0.0524 (4)	
H16	0.4634	0.1593	0.9404	0.063*	
C17	0.5384 (2)	0.22595 (10)	0.85088 (9)	0.0487 (4)	
H17	0.4301	0.2618	0.8426	0.058*	
C18	0.7349 (2)	0.04403 (12)	0.98061 (10)	0.0602 (4)	
C19	0.8407 (2)	0.39901 (10)	0.84592 (9)	0.0522 (4)	
H19A	0.9609	0.3843	0.8290	0.063*	
H19B	0.8212	0.3579	0.8860	0.063*	
C20	0.8583 (3)	0.48792 (11)	0.88137 (10)	0.0648 (5)	
H20A	0.9686	0.4897	0.9253	0.078*	
H20B	0.7419	0.5010	0.9018	0.078*	
C21	0.8851 (3)	0.55474 (11)	0.82047 (10)	0.0667 (5)	
H21A	0.8842	0.6117	0.8431	0.080*	
H21B	1.0093	0.5463	0.8046	0.080*	
C22	0.7218 (3)	0.54656 (11)	0.74953 (11)	0.0644 (5)	
H22A	0.7440	0.5860	0.7086	0.077*	
H22B	0.5997	0.5621	0.7645	0.077*	
C23	0.70745 (10)	0.45642 (4)	0.71727 (4)	0.0536 (4)	
H23A	0.5997	0.4530	0.6724	0.064*	
H23B	0.8260	0.4425	0.6987	0.064*	
O1	0.29677 (10)	0.39454 (4)	0.74661 (4)	0.0697 (4)	
H1	0.4335	0.4016	0.7749	0.094 (7)*	
N1	0.75228 (10)	−0.00682 (4)	1.02933 (4)	0.0807 (5)	
N2	0.67754 (10)	0.39314 (4)	0.77784 (4)	0.0440 (3)	

### Atomic displacement parameters (Å^2^)

**Table:**

	*U*^11^	*U*^22^	*U*^33^	*U*^12^	*U*^13^	*U*^23^
C1	0.0432 (8)	0.0496 (9)	0.0387 (8)	0.0036 (7)	0.0065 (6)	0.0061 (7)
C2	0.0494 (9)	0.0591 (10)	0.0518 (9)	0.0081 (7)	0.0131 (7)	0.0037 (8)
C3	0.0420 (9)	0.0822 (13)	0.0717 (12)	0.0114 (8)	0.0105 (8)	0.0137 (10)
C4	0.0477 (10)	0.0830 (13)	0.0578 (10)	−0.0052 (9)	−0.0029 (8)	0.0136 (10)
C5	0.0512 (9)	0.0594 (11)	0.0440 (8)	−0.0103 (8)	0.0023 (7)	0.0102 (8)
C6	0.0754 (12)	0.0720 (12)	0.0439 (9)	−0.0245 (10)	−0.0050 (8)	0.0024 (9)
C7	0.0973 (15)	0.0600 (12)	0.0518 (10)	−0.0129 (11)	0.0128 (10)	−0.0106 (9)
C8	0.0822 (13)	0.0589 (11)	0.0544 (10)	0.0052 (9)	0.0123 (9)	−0.0108 (9)
C9	0.0580 (10)	0.0560 (10)	0.0459 (9)	0.0041 (8)	0.0055 (7)	−0.0043 (8)
C10	0.0487 (8)	0.0459 (9)	0.0378 (8)	−0.0035 (7)	0.0062 (6)	0.0065 (7)
C11	0.0429 (8)	0.0429 (8)	0.0385 (7)	0.0048 (6)	0.0086 (6)	−0.0007 (6)
C12	0.0470 (8)	0.0397 (8)	0.0369 (7)	0.0018 (6)	0.0023 (6)	−0.0046 (6)
C13	0.0564 (10)	0.0612 (11)	0.0494 (9)	0.0161 (8)	0.0139 (7)	0.0094 (8)
C14	0.0642 (11)	0.0632 (11)	0.0571 (10)	0.0230 (9)	0.0108 (9)	0.0128 (9)
C15	0.0601 (10)	0.0470 (9)	0.0418 (8)	0.0005 (7)	0.0015 (7)	0.0024 (7)
C16	0.0587 (10)	0.0518 (10)	0.0476 (9)	−0.0017 (8)	0.0122 (7)	0.0020 (7)
C17	0.0518 (9)	0.0440 (9)	0.0504 (9)	0.0058 (7)	0.0099 (7)	0.0015 (7)
C18	0.0642 (11)	0.0609 (11)	0.0526 (10)	0.0036 (8)	0.0042 (8)	0.0089 (9)
C19	0.0622 (10)	0.0503 (9)	0.0417 (8)	−0.0038 (8)	0.0040 (7)	0.0016 (7)
C20	0.0842 (13)	0.0561 (11)	0.0537 (10)	−0.0122 (9)	0.0126 (9)	−0.0089 (8)
C21	0.0853 (13)	0.0460 (10)	0.0701 (11)	−0.0093 (9)	0.0186 (10)	−0.0056 (9)
C22	0.0784 (12)	0.0445 (10)	0.0725 (11)	0.0058 (8)	0.0204 (10)	0.0086 (9)
C23	0.0667 (10)	0.0486 (9)	0.0453 (9)	0.0064 (8)	0.0101 (7)	0.0076 (7)
O1	0.0583 (8)	0.0808 (9)	0.0726 (8)	0.0192 (6)	0.0193 (6)	−0.0094 (7)
N1	0.0778 (11)	0.0886 (12)	0.0752 (10)	0.0136 (9)	0.0138 (9)	0.0347 (10)
N2	0.0529 (7)	0.0407 (7)	0.0382 (6)	0.0048 (5)	0.0081 (6)	0.0009 (5)

### Geometric parameters (Å, °)

**Table:**

C1—C2	1.379 (2)	C14—C15	1.383 (2)
C1—C10	1.430 (2)	C14—H14	0.9300
C1—C11	1.528 (2)	C15—C16	1.384 (2)
C2—O1	1.3609 (2)	C15—C18	1.438 (2)
C2—C3	1.412 (2)	C16—C17	1.377 (2)
C3—C4	1.348 (2)	C16—H16	0.9300
C3—H3	0.9300	C17—H17	0.9300
C4—C5	1.406 (2)	C18—N1	1.1430 (18)
C4—H4	0.9300	C19—N2	1.4713 (16)
C5—C6	1.417 (2)	C19—C20	1.510 (2)
C5—C10	1.424 (2)	C19—H19A	0.9700
C6—C7	1.348 (3)	C19—H19B	0.9700
C6—H6	0.9300	C20—C21	1.515 (2)
C7—C8	1.386 (3)	C20—H20A	0.9700
C7—H7	0.9300	C20—H20B	0.9700
C8—C9	1.363 (2)	C21—C22	1.507 (2)
C8—H8	0.9300	C21—H21A	0.9700
C9—C10	1.415 (2)	C21—H21B	0.9700
C9—H9	0.9300	C22—C23	1.5069 (18)
C11—N2	1.4931 (2)	C22—H22A	0.9700
C11—C12	1.5163 (19)	C22—H22B	0.9700
C11—H11	0.9800	C23—N2	1.4793
C12—C13	1.384 (2)	C23—H23A	0.9700
C12—C17	1.386 (2)	C23—H23B	0.9700
C13—C14	1.376 (2)	O1—H1	0.9916
C13—H13	0.9300		
			
C2—C1—C10	118.69 (13)	C14—C15—C16	119.78 (14)
C2—C1—C11	121.54 (13)	C14—C15—C18	120.07 (15)
C10—C1—C11	119.74 (12)	C16—C15—C18	120.15 (15)
O1—C2—C1	123.11 (14)	C17—C16—C15	119.83 (15)
O1—C2—C3	116.13 (14)	C17—C16—H16	120.1
C1—C2—C3	120.75 (15)	C15—C16—H16	120.1
C4—C3—C2	121.00 (16)	C16—C17—C12	121.15 (14)
C4—C3—H3	119.5	C16—C17—H17	119.4
C2—C3—H3	119.5	C12—C17—H17	119.4
C3—C4—C5	120.90 (16)	N1—C18—C15	179.28 (18)
C3—C4—H4	119.6	N2—C19—C20	111.64 (13)
C5—C4—H4	119.6	N2—C19—H19A	109.3
C4—C5—C6	122.03 (16)	C20—C19—H19A	109.3
C4—C5—C10	118.90 (15)	N2—C19—H19B	109.3
C6—C5—C10	119.07 (16)	C20—C19—H19B	109.3
C7—C6—C5	122.08 (17)	H19A—C19—H19B	108.0
C7—C6—H6	119.0	C19—C20—C21	111.28 (14)
C5—C6—H6	119.0	C19—C20—H20A	109.4
C6—C7—C8	119.25 (17)	C21—C20—H20A	109.4
C6—C7—H7	120.4	C19—C20—H20B	109.4
C8—C7—H7	120.4	C21—C20—H20B	109.4
C9—C8—C7	121.01 (18)	H20A—C20—H20B	108.0
C9—C8—H8	119.5	C22—C21—C20	109.03 (15)
C7—C8—H8	119.5	C22—C21—H21A	109.9
C8—C9—C10	121.97 (16)	C20—C21—H21A	109.9
C8—C9—H9	119.0	C22—C21—H21B	109.9
C10—C9—H9	119.0	C20—C21—H21B	109.9
C9—C10—C5	116.63 (14)	H21A—C21—H21B	108.3
C9—C10—C1	123.65 (13)	C21—C22—C23	111.30 (13)
C5—C10—C1	119.71 (14)	C21—C22—H22A	109.4
N2—C11—C12	112.00 (10)	C23—C22—H22A	109.4
N2—C11—C1	111.23 (10)	C21—C22—H22B	109.4
C12—C11—C1	110.86 (11)	C23—C22—H22B	109.4
N2—C11—H11	107.5	H22A—C22—H22B	108.0
C12—C11—H11	107.5	N2—C23—C22	111.75 (7)
C1—C11—H11	107.5	N2—C23—H23A	109.3
C13—C12—C17	118.14 (14)	C22—C23—H23A	109.3
C13—C12—C11	120.24 (13)	N2—C23—H23B	109.3
C17—C12—C11	121.61 (13)	C22—C23—H23B	109.3
C14—C13—C12	121.40 (15)	H23A—C23—H23B	107.9
C14—C13—H13	119.3	C2—O1—H1	104.6
C12—C13—H13	119.3	C19—N2—C23	109.00 (7)
C13—C14—C15	119.67 (15)	C19—N2—C11	111.46 (9)
C13—C14—H14	120.2	C23—N2—C11	109.20 (6)
C15—C14—H14	120.2		
			
C10—C1—C2—O1	178.47 (12)	N2—C11—C12—C13	−114.13 (14)
C11—C1—C2—O1	0.41 (2)	C1—C11—C12—C13	121.01 (15)
C10—C1—C2—C3	−2.3 (2)	N2—C11—C12—C17	65.30 (16)
C11—C1—C2—C3	179.62 (14)	C1—C11—C12—C17	−59.56 (17)
O1—C2—C3—C4	−178.99 (15)	C17—C12—C13—C14	0.2 (2)
C1—C2—C3—C4	1.8 (3)	C11—C12—C13—C14	179.64 (15)
C2—C3—C4—C5	0.0 (3)	C12—C13—C14—C15	−1.0 (3)
C3—C4—C5—C6	178.39 (16)	C13—C14—C15—C16	0.3 (3)
C3—C4—C5—C10	−1.2 (2)	C13—C14—C15—C18	−178.43 (16)
C4—C5—C6—C7	−178.88 (17)	C14—C15—C16—C17	1.1 (2)
C10—C5—C6—C7	0.7 (2)	C18—C15—C16—C17	179.81 (14)
C5—C6—C7—C8	−0.7 (3)	C15—C16—C17—C12	−1.8 (2)
C6—C7—C8—C9	0.1 (3)	C13—C12—C17—C16	1.2 (2)
C7—C8—C9—C10	0.4 (3)	C11—C12—C17—C16	−178.23 (13)
C8—C9—C10—C5	−0.3 (2)	C14—C15—C18—N1	90 (16)
C8—C9—C10—C1	178.46 (15)	C16—C15—C18—N1	−89 (16)
C4—C5—C10—C9	179.41 (14)	N2—C19—C20—C21	−57.87 (19)
C6—C5—C10—C9	−0.2 (2)	C19—C20—C21—C22	54.4 (2)
C4—C5—C10—C1	0.6 (2)	C20—C21—C22—C23	−54.38 (19)
C6—C5—C10—C1	−179.01 (14)	C21—C22—C23—N2	57.88 (14)
C2—C1—C10—C9	−177.59 (15)	C20—C19—N2—C23	58.52 (13)
C11—C1—C10—C9	0.5 (2)	C20—C19—N2—C11	179.12 (12)
C2—C1—C10—C5	1.2 (2)	C22—C23—N2—C19	−58.58 (11)
C11—C1—C10—C5	179.26 (13)	C22—C23—N2—C11	179.46 (11)
C2—C1—C11—N2	−30.85 (18)	C12—C11—N2—C19	46.39 (14)
C10—C1—C11—N2	151.10 (12)	C1—C11—N2—C19	171.07 (11)
C2—C1—C11—C12	94.46 (16)	C12—C11—N2—C23	166.88 (8)
C10—C1—C11—C12	−83.59 (16)	C1—C11—N2—C23	−68.46 (10)

### Hydrogen-bond geometry (Å, °)

**Table:**

*D*—H···*A*	*D*—H	H···*A*	*D*···*A*	*D*—H···*A*
O1—H1···N2	0.99	1.70	2.614	151
C14—H14···N1^i^	0.93	2.55	3.395 (2)	151

Symmetry codes: (i) −*x*+2, −*y*, −*z*+2.
